# Operational research techniques applied throughout cancer care services: a review

**DOI:** 10.1080/20476965.2017.1414741

**Published:** 2018-01-17

**Authors:** Christina E. Saville, Honora K. Smith, Katarzyna Bijak

**Affiliations:** a Mathematical Sciences, University of Southampton, Southampton, UK.; b Southampton Business School, University of Southampton, Southampton, UK.

**Keywords:** Healthcare, operational research, review, cancer

## Abstract

Cancer is a disease affecting increasing numbers of people. In the UK, the proportion of people affected by cancer is projected to increase from 1 in 3 in 1992, to nearly 1 in 2 by 2020. Health services to tackle cancer can be grouped broadly into prevention, diagnosis, staging, and treatment. We review examples of Operational Research (OR) papers addressing decisions encountered in each of these areas. In conclusion, we find many examples of OR research on screening strategies, as well as on treatment planning and scheduling. On the other hand, our search strategy uncovered comparatively few examples of OR models applied to reducing cancer risks, optimising diagnostic procedures, and staging. Improvements to cancer care services have been made as a result of successful OR modelling. There is potential for closer working with clinicians to enable the impact of other OR studies to be of greater benefit to cancer sufferers.

## 1. Introduction

In the UK, the proportion of people affected by cancer is projected to increase from 1 in 3 in 1992, to nearly 1 in 2 by 2020 (Macmillan Cancer Support, 2013). National cancer services must deal with almost 340,000 new cancer cases yearly, in addition to continued care for patients previously diagnosed (Macmillan Cancer Support, 2014). The cancer care system is comprised of prevention, diagnosis, staging, and treatment services. Prevention includes screening a target population for cancers that are not yet showing symptoms (National Health Service, 2016) and encouraging lifestyle changes to lower cancer risk. Cancers are diagnosed following an abnormal screening result or development of suspicious symptoms. Following a cancer diagnosis, staging is a way of describing the size of the cancer and how much it has spread (Cancer Research UK, 2016). The stage and type of cancer affect which particular combination of treatments is most appropriate (National Cancer Institute, 2016a). There are a variety of treatments used in curing cancer, as well as adjuvant treatments to lower the risk of the cancer returning and palliative treatments to relieve symptoms (National Cancer Institute, 2016b).

Operational research (OR) is the “discipline of applying advanced analytical methods to help make better decisions” (The OR Society, 2016). The goal of this paper is to demonstrate the breadth of problems in cancer care that have been addressed with OR approaches. These methods have been successfully applied (and have the potential to help further) in medical decision-making for a variety of chronic diseases, as described in the literature reviews by Denton, Alagoz, Holder, and Lee (2011) and Capan et al. (2017). The latter review is particularly accessible to those less familiar with OR techniques. Further, the review by Steimle and Denton (2016) concentrates on applications of two particular OR methods, Markov decision processes and partially observable Markov decision processes, to prevention, detection, and treatment of chronic diseases. All of the aforementioned reviews contain cancer care examples, but unlike this review that is not their sole focus. Price, Golden, Wasil, and Denton (2016) review OR papers specifically addressing prostate cancer. There are also existing specialised reviews of OR work that focus on particular aspects of cancer care, which are described in the appropriate sections of this paper. However this review is, as far as we are aware, the first to provide examples of OR papers throughout cancer care services, without focussing on a particular cancer type.

In particular, we review OR papers addressing the different decisions encountered in cancer prevention, diagnosis, staging, and treatment services. We provide a sample of OR papers applied to decisions in each of these areas, rather than generating an exhaustive survey. The goals are to illustrate the types of cancer care-related questions that OR has helped to answer, as well as giving examples of different techniques that have been applied. This is a scoping rather than systematic review, since it is wide rather than deep (for example, if this were a systematic review, we would have listed all possible OR techniques as search terms). Consequently,our search will not have found all OR papers for cancer care, but it did yield a large sample of papers addressing a variety of questions throughout cancer care services.

We searched for relevant literature using the University of Southampton’s search engine DelphiS (University of Southampton, 2016). This covers a large number of databases including Scopus and JSTOR, which are two of the most widely used academic databases (Brailsford, Harper, Patel, & Pitt, 2009), as well as the biomedical and health database MEDLINE. We searched for “cancer” in abstracts, and “operations research” or “operational research” in the full text, yielding 1536 papers. We filtered this list by adding search terms (in abstracts) as follows: “screen${}^{*}$ OR prevent${}^{*}$” to find papers on prevention, “diag${}^{*}$ OR stag${}^{*}$” to find papers on diagnosis and staging papers, and “treat${}^{*}$” to find papers about treatment services. Additional papers found via other routes that met the goals of the review were also included. Papers were checked for relevance based on their abstracts; papers were deemed relevant if they used OR techniques to support decision-making in cancer care and the full text was accessible. Since we wish to show the range of problems addressed, we prioritise showing examples of different decision areas rather than many from the same area. That is why, for areas that have existing specialised literature reviews, we direct readers to these and provide only more recent examples. When finding recent examples of chemotherapy, surgery and radiotherapy papers we filtered the list using the following search terms: “chemo${}^{*}$”, “surg${}^{*}$”, “radio${}^{*}$” and “radiation”. This review describes 29 examples of OR applied to cancer prevention, 6 to diagnosis, 1 to staging and 44 to treatment.

**Table 1.** Examples of OR applied to cancer prevention.

Problem	Reference	Cancer type	Aim	Techniques
Reducing cancer risk	Hall et al. (1992)	Lung	Choosing which anti-smoking proposals to fund	Modified Delphi process, optimisation (binary integer program)
Reducing cancer risk	Kim et al. (2013)	Cervical	Analysing cost-effectiveness of HPV vaccination at country-level	Discrete time microsimulation and static cohort simulation
Locating screening facilities	Haase and Müller (2015)	Breast	Optimization of preventive health care facility locations	Multinomial logit model within linear optimisation
Locating screening facilities	Gu et al. (2010)	Breast	Optimization of preventive health care facility locations	Multi-objective optimization, heuristic
Evaluating process changes to screening services	Zai et al. (2014)	General	Evaluating the impact of introducing a screening invitation system	Discrete-event simulation (DES)
Evaluating process changes to screening services	Pilgrim and Chilcott (2008)	Cervical	Evaluating impact of process changes on reporting process	DES
Following up screening tests	Alagoz et al. (2013)	Breast	Optimising use of biopsies and follow-up mammograms	Bayesian network, Markov decision process (MDP)
Following up screening tests	Chhatwal et al. (2010)	Breast	Optimising use of biopsies	Bayesian network, MDP
Improving measurement of screening effectiveness	Vieira et al. (2011)	General	Comparing severity of tumours detected by screening compared to self-detected tumours	Discrete time simulation
Scheduling screening appointments	Baker and Atherill (2002)	Breast	Optimization of appointment schedule given attendance probability	Simulation-optimisation, heuristic

This paper proceeds as follows. We review OR papers addressing cancer prevention, diagnosis and staging, and treatment services in turn (see Tables 1–4). Within these sections, papers are grouped in terms of what problem they are addressing. When discussing the papers, we describe their goals, the techniques used and if present, any evidence of implementation. The performance measures of papers are summarised in Table 5. The findings of our review are presented in the “Conclusion and discussion” section, along with opportunities for further research.

**Table 2.** Examples of OR applied to cancer screening strategies.

Reference	Cancer type	Aim	Techniques
Arrospide et al. (2015)	Breast	Evaluation of screening strategy	Discrete event simulation (DES)
Ayer et al. (2012)	Breast	Optimising risk-based screening policy	Partially observable Markov decision process (POMDP)
Ayer (2015)	Breast	Finding sensitivity and screening values for which a screening policy is optimal	Partially observable Markov chain (POMC), non-linear program (inverse optimisation), heuristic algorithm
Ayer et al. (2016)	Breast	Optimising risk-based screening policy considering adherence	POMDP
Brailsford et al. (2012)	Breast	Comparing fixed-interval and age-based screening strategies considering adherence	DES, logistic regression
Madadi et al. (2015)	Breast	Comparing wide range of fixed-interval and age-based screening strategies considering adherence	POMC
O’Mahony et al. (2015)	Breast	Optimising risk-based screening policy	Mathematical model
Tejada et al. (2014)	Breast	Comparing fixed interval, risk-based and factor-based screening strategies	DES and system dynamics (SD)
Tejada et al. (2015)	Breast	Development of natural history of cancer model for use in above paper	DES and SD
Wang and Zhang (2017)	Breast	Optimising risk- and age-based screening policy	Logistic regression, misclassification cost criterion
Campbell et al. (2017)	Colorectal	Evaluating effect of screening of average risk individuals on colonoscopy resources required	DES
Erenay et al. (2014)	Colorectal	Optimising age-, gender-, and risk-dependent screening policy	POMDP
Hosking et al. (2013)	Colorectal	Comparing interventions to increase screening level	DES and SD
Li et al. (2014)	Colorectal	Comparing fixed-interval and observation-based screening strategies	POMC
Li et al. (2015)	Colorectal	Optimising age-, risk-dependent and observation-based screening policy considering adherence	POMDP
Song and Wang (2016)	Colorectal	Comparing fixed-interval and observation-based screening strategies	Monte Carlo simulation of Markov model
McLay et al. (2010)	Cervical	Optimising age-dependent screening policy	Simulation-optimisation
Rauner et al. (2010)	General chronic diseases, case study for breast cancer	Optimising fixed-interval screening strategies for different risk groups	Multi-objective optimisation, metaheuristic (Pareto ant colony optimisation)
Bertsimas et al. (2016)	Prostate	Finding optimal fixed-interval screening strategies according to multiple models	Multi-objective optimisation, local search heuristic

## 2. Cancer prevention

It is estimated that about half the cases of the 11 most common cancers are preventable (Soerjomataram, De Vries, Pukkala, & Coebergh, 2007). The risk of developing these cancers can be reduced by lifestyle changes such as stopping smoking, reducing alcohol intake, and exercising. According to Cancer Research UK (2016), some further cancer risk factors are as follows. Certain infections, for instance HPV and hepatitis B, can increase the risk of developing cancer. These are preventable through vaccination. Other risk factors are occupation-related or environmental, for example exposure to asbestos and sunlight. However there are risk factors for cancers that are not controllable; higher age and a family history of cancer both increase the chances of developing certain types of cancer. In recent years, genetic testing has started to be available to assess whether a person has inherited so-called cancer susceptibility genes (Macmillan Cancer Support, 2016). This has proved controversial because even if a person has a gene that signals they are at higher risk, this still does not mean they will definitely contract cancer.

At what stage of the disease a person is diagnosed can affect treatment decisions and ultimately survival chances. For this reason, screening programmes are offered for common cancers in an attempt to diagnose cases earlier, sometimes at the pre-cancerous stage (National Health Service, 2016). The harms and benefits of screening tests must be carefully balanced when deciding which screening method to use, who should be invited for screening and what the time interval between screening tests should be Sense about Science (2015). Screening methods are selected by trading-off cost and accuracy. The target population is chosen because it is predicted that they will benefit most, when taking into account harms. If the screening interval is too long, cases which develop between screens (interval cancers) will be missed. However screening too often increases harms such as increased exposure to radiation from mammograms.

First we discuss papers relating to reducing cancer risks, then screening strategies, followed by locating screening facilities. Next, papers on evaluating process changes to screening services and following up screening tests are reviewed. Finally, we describe two other screening-related studies, which address scheduling and the measurement of screening effectiveness. Tables 1 and 2 summarise key information about these papers.

### 2.1. Reducing cancer risks

Using our previously described search strategy, we found only two OR papers looking at cancer prevention through reducing cancer risks. Hall, Hershey, Kessler, and Stotts (1992) address smoking, a well-known risk factor for lung cancer, while Kim, Campos, O’Shea, Diaz, and Mutyaba (2013) are concerned with HPV vaccination, to prevent cervical cancer. We discuss these papers in turn.

Hall et al. (1992) investigate how best to choose between proposed projects to reduce the number of smokers in the USA. Stakeholders participated first in a modified Delphi process to determine appropriate decision criteria and then answered a questionnaire to decide on their relative importance. This led to development of a binary integer program which allocates funds between proposals based on budgetary constraints and how well the proposals meet the criteria. Using this approach, (Hall et al., 1992) were able to improve on the originally suggested selections of proposals, and it is reported that their recommendations were followed.

Kim et al. (2013) assess in which of 20 countries an HPV vaccination programme would be cost-effective, as well as modelling cytology-based screening in three countries where sufficient data were available. For this, they developed two simulation models: a population-level cohort simulation as well as an individual-level microsimulation. Results are given in terms of avoided cervical cancer cases and deaths, disability-adjusted life years (DALYs) and cost-effectiveness ratios. They showed that vaccination is cost-effective in most countries if the vaccine has a relatively low cost, but at higher costs, screening alone is more cost-effective in the three countries where this is considered.

### 2.2. Screening strategies

Screening strategies consist of who to invite, how often and which test to use. Evaluating and comparing screening strategies is a popular topic among OR and statistics researchers, with a series of review papers having been published covering this topic (Heidenberger, 1996; Knudsen, McMahon, & Gazelle, 2007; Pierskalla & Brailer, 1994; Stevenson, 1995). Most recently, Alagoz, Ayer, and Safa Erenay (2011) systematically reviewed those papers not described in detail previously. Screening models can be classified as either simulation or analytical. Both are powerful techniques but have downsides; simulations can only compare a relatively small number of scenarios, whereas analytical models tend to make some unrealistic simplifying assumptions (Alagoz et al., 2011). Furthermore, Alagoz et al. (2011) stress the importance of using reliable data to develop models. Koleva-Kolarova, Zhan, Greuter, Feenstra, and De Bock (2015) provide a recent review of papers using simulation to assess breast cancer screening strategies in particular. Here the focus is on models that feature in multiple publications. These models have been used to influence screening decisions in different settings, although (Koleva-Kolarova et al., 2015) warn that they have not been validated with data outside the setting for which they were originally developed.

Given the vast body of literature in this area, we here only provide examples published since Alagoz et al. ’s (2011) review. Table 2 displays key information about such papers that were uncovered by our search: aims, techniques, and cancer types. We next discuss further details about these papers, starting with those addressing breast cancer, then colorectal cancer and finally other or unspecified cancers.

#### 2.2.1. Breast cancer screening strategies

The majority of breast cancer screening papers either compare mammogram screening strategies (Brailsford, Harper, & Sykes, 2012; Madadi, Zhang, & Henderson, 2015; Tejada et al., 2014) or optimise the decision of whether to mammogram or not each year (Ayer, Alagoz, & Stout, 2012; O’Mahony et al., 2015; Ayer, Alagoz, Stout, & Burnside, 2016; Wang & Zhang, 2017). Of these, Madadi et al. (2015) consider an especially large set of screening strategies. There has been a shift in focus away from the fixed-interval policies that are common in practice, to consideration of dynamic screening intervals (Ayer et al., 2012; Brailsford et al., 2012; Tejada et al., 2014; Madadi et al., 2015; O’Mahony et al., 2015; Ayer et al., 2016; Wang & Zhang, 2017). These dynamic intervals may vary based on changing risk, age and adherence to screening guidelines. Adherence is modelled in a range of ways from detailed psychological models of behaviour (Brailsford et al., 2012), to changing physician belief about which patients are regular or irregular screeners (Ayer et al., 2016), and to uncertain adherence probabilities based on age and screening interval (Madadi et al., 2015). Arrospide et al. (2015), on the other hand, aim to evaluate how well a particular screening strategy is likely to perform in the long run based on short term real-world results. When choosing a screening method, there is a trade-off between sensitivity, the probability of screening correctly identifying a person with cancer, and specificity, the probability of screening correctly identifying a person without cancer. Ayer (2015) assess for what range of sensitivity and specificity values a screening interval is best. If more accurate screening tests are introduced in future, the ongoing appropriateness of the screening interval can be judged by this model.

Both simulation (Arrospide et al., 2015; Brailsford et al., 2012; Tejada et al., 2014, 2015) and analytical (Ayer et al., 2012; Ayer, 2015; Ayer et al., 2016; Madadi et al., 2015; O’Mahony et al., 2015; Wang & Zhang, 2017) techniques are used to model breast cancer progression and the screening process, as in earlier papers. There are some very sophisticated models combining multiple approaches, for example Tejada et al. (2014; 2015) combine system dynamics with detailed discrete event simulations. Contrastingly, O’Mahony et al. (2015) purposely built a relatively simple mathematical model, validated its results against a more complex simulation and found that their model was detailed enough to demonstrate that different risk levels have different optimal screening intervals. The performance measures assessed by the models are diverse and include mortality measures (Arrospide et al., 2015; Brailsford et al., 2012; Madadi et al., 2015; Tejada et al., 2014; Wang & Zhang, 2017), quality-adjusted life year measures (Ayer et al., 2012; Ayer, 2015; Ayer et al., 2016; Madadi et al., 2015; Tejada et al., 2014) and cost-effectiveness measures (O’Mahony et al., 2015; Tejada et al., 2014).

#### 2.2.2. Colorectal screening strategies

Screening methods for colorectal cancer include faecal occult blood test (FOBT) and colonoscopy. Hosking, Roberts, Uzsoy, and Joseph (2013), Erenay, Alagoz, and Said (2014) and Li, Zhu, Klein, and Kong (2014) focus on colonoscopy only screening strategies, while Li, Dong, Ren, and Yin (2015), Song and Wang (2016) and Campbell, Blake, Kephart, Grunfeld, and MacIntosh (2017) consider two-step strategies that follow a non-invasive test with a colonoscopy for those patients that need it. The majority of these papers compare screening strategies (Li et al., 2014; Song & Wang, 2016) or optimise the yearly screening decision (Erenay et al., 2014; Li et al., 2015). In all cases, dynamic screening intervals are evaluated, including so-called “observation-based” strategies (Li et al., 2014; Li et al., 2015; Song & Wang, 2016), which use the results of the previous screen to inform the next screening interval. Li et al. (2015) consider patients with a range of adherence probabilities. A different problem is tackled by Campbell et al. (2017), who investigate the effect of screening the general population on colonoscopy resources available to other groups, such as surveillance and high risk (see also the paper by Güneş, Örmeci, and Kunduzcu, 2015 discussed in the next section). Hosking et al. (2013) instead look at how to increase the proportion of the population screened through both supply and demand interventions.

Similar techniques are used for colorectal screening as for mammography: simulation (Campbell et al., 2017; Hosking et al., 2013; Song & Wang, 2016) and analytical (Erenay et al., 2014; Li et al. ; 2015). A combination of discrete event simulation and system dynamics is applied to understand the likely impacts of different interventions hoped to increase the screening level (Hosking et al., 2013). Both papers optimising the colorectal screening decision each year use partially observable Markov decision processes (Erenay et al., 2014; Li et al., 2015). Screening strategies are compared in terms of quality-adjusted life years in most cases (Erenay et al., 2014; Li et al., 2014; Li et al., 2015). Li et al. (2014) also consider total cost, while Song and Wang (2016) derive a new cost-effectiveness measure.

#### 2.2.3. General cancer and other cancer screening strategies

Finally, we discuss papers addressing screening models for other cancers, as well as general screening models. One paper is about cervical cancer screening through pap smears (McLay, Foufoulides, & Merrick, 2010), one is for general cancer screening with an application to prostate cancer screening (Bertsimas, Silberholz, & Trikalinos, 2016) and the other is a model for general chronic diseases, with a case study for breast cancer screening (Rauner, Gutjahr, Heidenberger, Wagner, & Pasia, 2010). McLay et al. (2010) and Rauner et al. (2010) both develop new models to optimise screening policies, while Bertsimas et al. (2016) combine recommendations from multiple existing models to find screening strategies that balance being optimal on average with those being optimal according to the most pessimistic model. Both Bertsimas et al. (2016) and Rauner et al. (2010) limit the policies under consideration to fixed interval, but McLay et al. allow the screening interval to vary with age. A strength of Rauner et al. ’s (2010) model is that it can be used to optimise screening strategies across several diseases simultaneously.

Different techniques are used than in the breast and colorectal papers described above. McLay et al. (2010) develop a simulation-optimisation model, while both Rauner et al. (2010) and Bertsimas et al. (2016) formulate multi-objective optimisation problems that are solved with metaheuristics and a local search heuristic, respectively. McLay et al. (2010) consider three different performance measures (number of cervical cancer deaths, number of life years lost due to cervical cancer and cervical cancer incidence), and optimise separately for each of them. Rauner et al. (2010) and Bertsimas et al. (2016) assess models in terms of quality-adjusted life years; Rauner et al. (2010) also set a budget constraint.

### 2.3. Locating screening facilities

Gu, Wang, and McGregor (2010) and Haase and Müller (2015) aim to determine the best locations for screening facilities. Both case studies involve locating breast cancer screening services. Haase and Müller (2015) extend previous work by Zhang, Berman, and Verter (2012) who incorporated a discrete choice model inside an optimisation. Haase and Müller (2015) reformulate this non-linear model so that it is linear and able to solve mid-size instances to optimality or close to optimality within one hour. Gu et al. (2010), on the other hand, solve their multi-objective optimisation with a heuristic, which consists of trying to improve upon the position of each facility one at a time.

The authors measure the suitability of location sets in different ways. Haase and Müller (2015) fix a minimum demand at each centre in order to ensure quality, and choose the location set that maximises participation in screening. However Gu et al. (2010) maximise both the efficiency, which is a measure of fairness and is the sum of weighted accessibility values, and the coverage, which measures how many people are within an acceptable distance of a facility. In their case study, the optimum solution improves both the efficiency and coverage with fewer facilities compared to the current set-up.

### 2.4. Evaluating process changes to screening services

Pilgrim and Chilcott (2008) and Zai et al. (2014) both evaluate process changes to screening services. In particular, Pilgrim and Chilcott (2008) model the process for reporting cervical smear test results, from the time of the smear test until patients receive results. Zai et al. (2014) instead measure the long-term effect of introducing a screening appointment system, by modelling the IT workflow and staff responsible for communicating reminders. Both studies employ discrete event simulation, which allows the authors to experiment with different changes to the current processes. The aim of Pilgrim and Chilcott ’s (2008) work is to find ways to reduce the length of time patients wait for results while Zai et al. ’s (2014) performance measure is the average number of overdue screenings per patient.

Pilgrim and Chilcott (2008) found that liquid-based cytology should reduce the time patients wait for results, and with some additional minor changes, could reach 95% receiving results within two weeks, as well as being cost-saving in the long run. Their results were communicated to UK Government ministers and so informed thinking about policy changes. The simulation developed by Zai et al. (2014) predicted that the invitation system would reduce overdue screenings per patient compared to the current process, in the long run. Perhaps unsurprisingly, they also found that increasing intervals between screens and increasing numbers of staff involved with the invitation process both reduced overdue screenings.

### 2.5. Following up screening tests

Two related papers address how patients should be managed following abnormal mammogram results (Chhatwal, Alagoz, & Burnside, 2010; Alagoz, Chhatwal, & Burnside, 2013). The aim is to identify those patients at high enough risk of breast cancer to justify the expense, worry and possible harm caused by carrying out further tests. The risk of cancer is calculated from mammogram results and demographic data using a Bayesian network (Chhatwal et al., 2010), and these risk scores are also used by Alagoz et al. (2013). Both papers formulate the problem as a Markov decision process and aim to maximise total expected quality-adjusted life years. Chhatwal et al. (2010) optimise the decision of whether or not to biopsy, while Alagoz et al. (2013) additionally consider follow-up mammograms as an option. Chhatwal et al. (2010) find that the risk threshold at which patients should be biopsied depends on age. According to Alagoz et al. ’s (2013) model, fewer biopsies and follow-up mammograms should be carried out than were recommended by radiologists.

### 2.6. Other screening-related studies

Here we discuss two further examples of OR work related to cancer screening. Firstly, Baker and Atherill (2002) schedule screening appointments for breast cancer. They predict individual no-show probabilities based upon previous attendance behaviour, then develop a simulation model and a heuristic procedure to optimise a combination of waiting time, idle time, and overtime. It was found that screening sessions should start with the patients who are most likely to attend, and end with some overbooked appointments. This is predicted to increase throughput by 10%.

Secondly, Vieira, de Senna, Harper, and Shahani (2011) aim to improve how screening effectiveness is measured. In particular, they compare the severity of breast cancer detected by screening to self-detected breast cancers. This is achieved by fitting distributions to data on tumour size and developing a simulation model of tumour progression, screening and self-detection. The output is tumour doubling times for screen-detected and self-detected cancers. It was found that the increased survival benefits achieved through screening for cancer at fixed intervals have been overestimated, due to so-called “length bias”. This means that slower growing tumours are more likely than faster growing tumours to be detected at the time of screening, rather than being self-detected in between screens.

## 3. Cancer diagnosis and staging

Cancers are diagnosed following an abnormal screening result, development of suspicious symptoms or incidental detection when examining patients for other reasons, for example after being admitted through the emergency department or during an unrelated medical consultation (Elliss-Brookes et al., 2012). After screening, patients with abnormal results are typically invited for further diagnostic tests to confirm or exclude a cancer diagnosis. In the UK, patients who self-detect suspicious symptoms are encouraged to visit their GP who will refer them to a specialist diagnostic clinic if necessary. Patients who have been diagnosed with cancer may undergo further tests to determine the size of the cancer and how much it has spread (Cancer Research UK, 2016). This staging process helps to determine the most appropriate treatment.

Any models about cancer diagnosis whose main focus is screening asymptomatic patients were described in the previous section. Other OR applications to cancer diagnosis, as well as staging, are described in this section. Note that there are also papers predicting a patient’s risk or stage of cancer in the related areas of data mining and statistics, which we do not discuss here (see for example Hippisley-Cox and Coupland, 2013a; 2013b). We discuss papers on managing diagnostic resources, then optimising diagnostic tests and finally staging accuracy. Summaries of these papers are displayed in Table 3.

**Table 3.** Examples of OR applied to cancer diagnosis and staging.

Problem	Reference	Cancer type	Aim	Technique
Managing diagnostic resources	Lee et al. (2014)	General	What is optimal schedule for producing and delivering nuclear medicine to hospitals?	Mixed-integer program, metaheuristics
Managing diagnostic resources	Güneş et al. (2015)	Colorectal	What capacity of colonoscopy resources should be allocated to screening and diagnosis?	Compartmental models, system dynamics (SD)
Managing diagnostic resources	Örmeci et al. (2016)	Colorectal	How to dynamically prioritise screening and diagnostic colonoscopies	Markov decision process, event-based dynamic programming
Managing diagnostic resources	Berg et al. (2010)	Colorectal	Testing potential impact of changes to resource use	discrete event simulation (DES)
Managing diagnostic resources	Berg et al. (2013)	Colorectal	Comparing overbooking and strategies to reduce no-shows on clinic performance	DES
Optimising diagnostic procedure	Sofer et al. (2003)	Prostate	Optimising biopsy positions	Non-linear integer program, generalised decomposition algorithm
Staging accuracy	Ekaette, Lee, Kelly, and Dunscombe (2006)	Breast	What is the chance of mis-staging patients and providing wrong treatment?	Monte Carlo simulation

### 3.1. Managing diagnostic resources

This subsection describes some papers on how best to manage diagnostic resources. We discuss papers on scheduling production and delivery, allocating resources and evaluating service improvements in turn.

Nuclear medicine can aid in the diagnosis and monitoring of cancer. Lee, Kim, Johnson, and Lee (2014) formulate a mixed-integer program of the production and delivery schedule for nuclear medicine, given the time limit on its effectiveness. They apply a metaheuristic consisting of five algorithms to find solutions with low cost. For example, in their case study, they are able to reduce the number of vehicles needed by between two and three. This problem is in some respects similar to the problem solved by Chahed, Marcon, Sahin, Feillet, and Dallery (2009), which is described in the “Access to treatment” section.

Güneş et al. (2015) address the tactical decision of how best to allocate colonoscopy resources between screening and symptomatic patients. Relatedly, Örmeci, Günes, and Kunduzcu (2016) model the operational decision of how to dynamically prioritise screening and symptomatic patients. These are both somewhat similar to the paper by Campbell et al. (2017) described previously in the “Colorectal screening strategies” section, but they use different techniques and address slightly different questions. Güneş et al. (2015) develop compartmental models, where each compartment contains patients in the same disease stage and the same health service stage. Differential equations calculate the rate at which the population progress through these stages. Using a system dynamics simulation, the authors find that less capacity should be allocated to screening if the mortality rate is more important than the incidence rate. On the other hand, Örmeci et al. (2016) develop a Markov decision process model and apply event-driven dynamic programming. Since preventive services lower the demand for symptomatic colonoscopies, the authors show that for certain parameters it would be optimal (from a cost perspective) to prioritise screening. However when applying their model to real data on colonoscopy services, they find that symptomatic patients should be prioritised.

Berg et al. (2010, 2013) also investigate colonoscopy procedures, but unlike Güneş et al. (2015), they use discrete event simulation. Berg et al. (2010) experiment with changing the staff-to-room ratio, appointment times and turnaround time. On the other hand, Berg et al. (2013) investigate the effect of overbooking versus alternative strategies to reduce missed appointments. In terms of assessing performance, Berg et al. (2010) consider throughput and a range of resource utilisation measures. Berg et al. (2013) combine operating costs, waiting time, and overtime costs into a single performance measure that represents expected net gain. Berg et al. (2010) recommend that there should be at most two rooms per endoscopist and that the gap between patient appointments should be increased by five minutes, which would decrease waiting time without lengthening the clinic day. Berg et al. (2013) find that overbooking is the most successful strategy.

### 3.2. Optimising diagnostic procedures

Sofer, Zeng, and Mun (2003) optimise how biopsies are used to detect prostate cancer. In particular, they analyse prostate specimens to find out how likely cancer is to affect different parts of the prostate. Then they formulate a non-linear program to find the best needle locations to maximise the probability of detecting cancer in affected patients. Their method accounts for randomness in the exact positioning, depth and angle of the biopsy needle. The authors were able to improve the chance of detecting cancer compared to the current biopsy positioning rules, without increasing the number of samples taken.

### 3.3. Staging accuracy

Ekaette et al. (2006) use a Monte Carlo simulation to model staging and radiation treatment of post-surgery breast cancer patients. The aim is to determine how often a mistake is made in determining the stage of cancer, leading to patients not receiving the most effective treatment. Additionally, the expected information value of each combination of tests in identifying metastases (secondary tumours) is calculated. It is found that there is a small chance of patients being misstaged and hence receiving the wrong type of radiation treatment.

## 4. Cancer treatment

The appropriate treatment or combination of treatments depends on the type of cancer and stage. Additionally, personal preferences regarding convenience and potential side effects also affect choice of treatment. Many cancers are treated by performing surgery to remove the tumour, and other common treatments are chemotherapy and radiotherapy (Macmillan Cancer Support, 2016). Another treatment is photodynamic therapy which involves drugs called photosensitisers that are activated by light. Cancer patients may need to attend clinics regularly for treatment or be supplied with medicines in their homes, so planning access to treatment is important. Cancer treatment centres are concerned with how best to organise their services to meet performance measures, and indeed what these performance measures should be.

**Table 4.** Examples of OR applied to cancer treatment.

Problem	Reference	Focus	Aim	Technique
Treatment decision	Utley et al. (2006)	Non-small cell lung cancer	Calculating survival benefit of post-operative chemotherapy on patients with different stages of cancer	Mathematical modelling involving a proportional hazard model
Treatment decision	Suner et al. (2012)	Renal cancer	Develop a decision support for primary and additional treatments based on differing expert opinions	Analytic hierarchy process (AHP), sequential decision tree
Treatment decision	Simon (2009)	Prostate cancer	Developing tool for patients to choose between treatments	Decision analysis
Access to treatment	Cotteels et al. (2012)	Radiotherapy	Optimising locations of treatment centres	Optimisation, p-median method
Access to treatment	Chahed et al. (2009)	Chemotherapy	What is the optimal schedule for producing and delivering chemotherapy to patients at home?	Travelling salesman and scheduling, branch and bound
Performance of cancer treatment centres	Santos et al. (2007)	Radiotherapy	Designing appropriate performance criteria	System dynamics (SD) and multi-criteria decision analysis
Performance of cancer treatment centres	Baesler and Sepúlveda (2001)	Chemotherapy	How many resources (treatment chairs, nurses, laboratory staff and equipment, and pharmacy staff and equipment) are required?	Goal programming simulation-optimisation, genetic algorithm
Performance of cancer treatment centres	Matta and Patterson (2007)	Chemotherapy and radiotherapy	Compare strategies to improve performance of treatment centre	Discrete event simulation (DES)
Performance of cancer treatment centres	Werker et al. (2009)	Radiotherapy planning	Compare strategies to reduce treatment planning time	DES
Scheduling	Mutlu et al. (2015)	Breast surgery	Optimising multidisciplinary team schedules	Integer program, simulation
Scheduling	Lim et al. (2016)	Surgery	Optimise assignment of nurses to surgery cases and optimise lunch breaks	Multi-objective optimisation (mixed-integer program), swap heuristic, column generation approach
Scheduling	Mobasher et al. (2011)	Surgery	Optimise assignment of nurses to surgery cases	Multi-objective optimisation (mixed-integer program), a new version of modified goal programming, solution pool method
Scheduling	Vanberkel et al. (2011)	Surgery	Comparing impact of different surgical block schedules on workload in other departments	Analytical models involving queuing theory
Scheduling	Hahn-Goldberg et al. (2014)	Chemotherapy	Optimising patient appointment times within a day	Constraint programming optimisation, “shuffle” algorithm
Scheduling	Santibáñez et al. (2012)	Chemotherapy	Comparing changes to booking process, optimising patients appointment times and evaluating real impact of service changes	DES, multi-objective optimisation (integer program)
Scheduling	Woodall et al. (2013)	Chemotherapy	Optimising nurse shift start times	DES, optimisation
Scheduling	Bikker et al. (2015)	External radiotherapy	Optimising doctors’ allocation to pre-treatment appointments	Integer program, DES
Scheduling	Petrovic, Morshed, et al. (2011)	External radiotherapy	Optimising treatment start days	Multi-objective optimisation, genetic algorithm
Scheduling	Castro and Petrovic (2012)	External radiotherapy	Optimising pre-treatment appointments	Multi-objective optimisation (mixed-integer programs), problems solved hierarchically
Scheduling	Conforti et al. (2010)	External radiotherapy	Optimising treatment start days	Integer program
Scheduling	Legrain et al. (2015)	External radiotherapy	Optimising treatment start days	Stochastic optimisation, greedy and primal-dual algorithms
Scheduling	Sauré et al. (2012)	External radiotherapy	Optimising treatment start days	Markov decision process, approximate dynamic programming
Treatment planning	Alam et al. (2013)	Chemotherapy	Optimising treatment plan	Multi-objective optimisation, closed-loop optimal control model, genetic algorithm
Treatment planning	Lee and Zaider (2008)	Low-dose rate brachytherapy, prostate cancer	Optimising placement of radioactive seeds in real-time	Mixed-integer program, conflict hypergraphs for dealing with dense constraint matrices
Treatment planning	Ferrari et al. (2014)	Low-dose rate brachytherapy, prostate cancer	Optimising placement of radioactive seeds pre-operation	Mixed-integer program, Genetic algorithm
Treatment planning	Lee et al. (2013)	High-dose rate brachytherapy, cervical cancer	Optimising position of radioactive sources and dwell time	Mixed-integer non-linear program, branch-and-cut and local search involving generalised conflict hypergraphs
Treatment planning	Holm et al. (2016)	High-dose rate brachytherapy, prostate cancer	Optimising position of radioactive sources and dwell time	Mixed-integer program, tabu search, variable neighbourhood search, genetic algorithm
(*Continued*)				

**Table 5.** (*Continued*).

Problem	Reference	Focus	Aim	Technique
Treatment planning	Teodorović et al. (2013)	Radioactive iodine therapy, thyroid cancer	Replicating experienced physicians’ dose plans	Case-based reasoning, Bee Colony Optimisation meta-heuristic
Treatment planning	Petrovic et al. (2016)	3D conformal, brain cancer	Designing treatment plans from previous cases	Case-based reasoning, adaptation approaches
Treatment planning	Jalalimanesh et al. (2017)	Intensity-modulated radiation therapy (IMRT)	Optimising number of treatments (fractions) and doses (fraction size)	Agent-based simulation, reinforcement learning
Treatment planning	Dias et al. (2014)	IMRT	Optimising beam angles	Genetic algorithm and neural network
Treatment planning	Mahmoudzadeh et al. (2016)	IMRT, breast cancer	Optimising beamlet intensities under breathing uncertainty	Robust optimisation, conditional value-at-risk, decomposition (constraint generation)
Treatment planning	Obal et al. (2013)	3D conformal radiotherapy, prostate cancer	Optimising dose per beam	Multi-objective optimisation (linear program), weighted sum method
Treatment planning	Petrovic, Mishra, et al. (2011)	3D conformal radiotherapy, prostate cancer	Optimising total dose in two phases of treatment	Case-based reasoning, simulated annealing
Treatment planning	Van Haveren et al. (2017)	IMRT, prostate cancer	Optimising beamlet intensities	Multi-objective (convex) optimisation, lexicographic reference point method
Treatment planning	Chan et al. (2014)	IMRT, breast cancer	Optimising beamlet intensities under breathing uncertainty	Robust optimisation, conditional value-at-risk
Treatment planning	Chan and Mišić (2013)	IMRT, lung cancer	Optimising beamlet intensities under breathing uncertainty	Robust optimisation, series of linear programs
Treatment planning	Aleman et al. (2014)	IMRT, head and neck cancer	Optimising beamlet intensities considering two different tumour sites	Multi-objective (convex) optimisation, interior point method
Treatment planning	Cabrera et al. (2014)	IMRT	Optimising beamlet intensities	Multi-objective optimisation
Treatment planning	Bertsimas, Cacchiani, Craft, and Nohadani (2013)	IMRT, pancreatic cancer	Optimising beamlet intensities and beam angles	Linear program, simulated annealing combined with gradient descent
Treatment planning	Taşkin and Cevik (2013)	IMRT	Optimising leaf sequencing	Mixed-integer program, combinatorial Benders decomposition
Chemotherapy drug production policy	Masselink et al. (2012)	Chemotherapy	Deciding which chemotherapy drugs to prepare in advance	Analytical models involving queuing theory, DES
Chemotherapy drug production policy	Vidal et al. (2010)	Chemotherapy	Deciding which chemotherapy drugs to prepare in advance	AHP
Optimising treatment	Holder and LLagostera (2008)	Photodynamic therapy	Modelling effects of treatment	Linear program, interior-point algorithm

Cancer surgery may remove all or part of the tumour. Patients undergoing surgery will likely need to spend some time in hospital afterwards to recover, which means there is an interaction between operating room workload and workload in inpatient wards. Multidisciplinary teams may be needed to perform these complex surgeries, which means scheduling should take into account the availability of different professionals.

Chemotherapy requires patients to take drugs that are designed to destroy cancer cells. Unfortunately, healthy cells may also be damaged, and some of the drugs have harmful side effects. Patients taking the drugs orally may stay at home, but when the treatment is fed into a vein, patients typically visit an outpatient clinic (Cancer Research UK, 2016). Outpatient chemotherapy visits are complicated to schedule because patients receive this treatment in cycles, where the gap between visits is different for different patients. Since pharmacists prepare drugs specially for each patient, drugs may go to waste if patients are too ill for treatment.

Radiation treatment may be internal or external. There is a balance between providing a high enough dose to cancer cells while keeping the dose reaching the surrounding normal tissues and organs low. Internal radiation treatment consists of radioactive sources being placed inside the body (Macmillan Cancer Support, 2016). When the radioactive sources are solid, this is known as brachytherapy, which can be delivered in low or high doses, through permanent or temporary implants. In radioisotope therapy, radiation is delivered through injections, capsules, or drinks.

External radiotherapy consists of targeting the cancer site with X-ray beams using a linear accelerator (LINAC) machine (Cancer Research UK, 2016). Patients must undergo a pre-treatment assessment involving imaging, for the specialists to decide the area to target, the appropriate dosage and how to configure the LINAC. 3D conformal radiotherapy uses multileaf collimators to block parts of the beams to match the shape of the tumour. Intensity-modulated radiotherapy (IMRT) is a more advanced method where the beams are divided into beamlets with different intensities.

This section proceeds as follows. First, we discuss OR models relating to treatment decisions, then access to treatment followed by performance of cancer treatment centres. Then we address scheduling of cancer surgery, chemotherapy and radiotherapy. Chemotherapy planning and different types of radiotherapy planning are addressed next. We finish the section with other treatment-related studies (deciding which drugs to produce in advance, and planning photodynamic therapy). The papers are summarised in Table 4.

### 4.1. Treatment decisions

There are examples of OR techniques helping with patients’ cancer treatment decisions (Simon, 2009; Suner, Çelikoğlu, Dicle, & Sökmen, 2012; Utley, Paschalides, & Treasure, 2006). These studies are about various different cancer types and treatment options. Utley et al. (2006) focus on whether patients who have had surgery that appears to have cured their non-small cell lung cancer should have chemotherapy afterwards. Simon (2009) addresses the choice of prostate cancer treatments, while Suner et al. (2012) model the two sequential decisions of renal cancer treatments. A range of techniques are applied. Utley et al. (2006) develop a mathematical model based on proportional hazards, in order to calculate the survival benefit of chemotherapy depending on its hazard ratio and the patient’s stage of disease. The outcome of the model is the average additional months survival over a five-year period, and ranges from zero to twelve months. Contrastingly, Suner et al. (2012) use the analytic hierarchy process to merge five experts’ opinions on the importance of criteria in making two sequential treatment decisions. They find that the judgements of individuals are consistent, and represent the results in decision tree format. Finally, Simon ’s (2009) decision tool asks patients to quantify the expected influence of each treatment side effect on their quality of life. These are combined with the survival impacts of treatments and side effects to produce a “life score” for each treatment. This tool is available online.

### 4.2. Access to treatment

Here, we describe OR approaches to improving access to cancer treatment or medicines. Firstly, Cotteels, Peeters, Coucke, and Thomas (2012) consider the problem of locating radiotherapy centres in Belgium. They use the *p*-median method, which minimises the total demand-weighted distance between each patient and the nearest radiotherapy centre, given that there are *p* centres. The locations recommended by the model are compared to the actual locations, in order to highlight inequities in provision. They found that the current locations are for the most part near the locations suggested by the model.

Furthermore, Chahed et al. (2009) address the problem of efficiently supplying chemotherapy drugs to patients in their homes. A travelling salesman model is extended to incorporate scheduling of the preparation of drugs, which each have a production duration, administration duration, and expiry date. This is somewhat similar to the paper by Lee et al. (2014) that was described in the “Managing diagnostic resources” section, since it also deals with drugs, with expiry dates, that must be produced and supplied effectively. There are some differences including that in Chahed et al. ’s (2009) paper the nurse who delivers the drugs stays with the patient to administer them, and in the mathematical formulation only one nurse travelling one route is considered. Using branch and bound to solve for the minimum distance, the authors find that depending on the parameters, there may be no solution, the same solution as if only routing were considered, or a different solution.

### 4.3. Performance of cancer treatment centres

In this section, we discuss papers that address how well cancer treatment centres perform. According to Brailsford and Vissers (2011), two stages of developing and managing health services relate to performance: defining performance criteria and managing how well these are met. Some performance measures can be improved by changing the scheduling of patients or resources. OR approaches to optimal scheduling of chemotherapy and radiotherapy services are discussed separately in following sections.

Santos, Belton, and Howick (2007) focus on designing appropriate performance measures for a radiotherapy department. It is reported that the current performance measurement is inadequate because only a few aspects are considered and there is no systematic regular process for measuring performance. The authors use system dynamics and multi-criteria analysis to identify summary indicators (relating to capacity, access, efficiency, and outcomes) that better represent overall performance, help clients understand some underlying causes for the observed performance and predict the impact of changes on performance.

Baesler and Sepúlveda (2001), Matta and Patterson (2007) and Werker, Sauré, French, and Shechter (2009) all focus on how cancer treatment centre performance can be improved. Matta and Patterson (2007) present a framework for combining multiple performance measures across multiple dimensions into a single score. They develop a discrete event simulation model of a cancer treatment centre offering both chemotherapy and radiotherapy. For this case study, the performance score consists of the average system time and average overtime weighted by throughput and frequency, respectively, and stratified by day of week, disease type, and patient routing through facilities. A variety of process, resource, and scheduling changes are simulated and compared in terms of the performance score. In this way, 11 changes that individually improved overall performance were identified and then their joint impact on performance was assessed. As a result of the study, the treatment centre has changed how appointments are scheduled, increased capacity, and introduced a separate blood testing area.

Baesler and Sepúlveda (2001) use a goal programming simulation-optimisation method to find the numbers of different resources required in a chemotherapy centre. Multiple objectives are considered (waiting time, chair utilisation, closing time, and nurse utilisation), and weighted based on their importance. A genetic algorithm is used to find possible solutions to the problem and succeeds in finding a configuration of resources that is at least as good as the current configuration in all four objectives. In particular, it improves nurse utilisation and needs just one extra chair.

Werker et al. (2009) also develop a discrete event simulation. Specifically they model the radiation therapy pre-treatment process in order to find ways to reduce its length (the total planning time). This consists of multiple stages required to plan treatment including consultations, oncologist input, dose planning, and verification stages. Three different types of staff are involved in the planning: oncologists, radiation therapists and medical physicists. It was found that shorter, more consistent delays to oncologists being available would reduce the total planning time.

### 4.4. Surgery scheduling

Mutlu, Benneyan, Terrell, Jordan, and Turkcan (2015) optimise the individual schedules of members of a multidisciplinary team in order to maximise the time that they are available to work together. They formulate the problem as an integer program that includes restrictions relating to preferences and availability for clinic work. Their case study, optimising the schedules of plastic and oncologic surgeons, succeeded in increasing the number of sessions when teams of two surgeons were available for breast cancer surgery by 94%.

Vanberkel et al. (2011), on the other hand, compare surgical block schedules, that is, which blocks of operating room time are assigned to different specialties. They use an analytic approach involving queuing theory to output the workload in different wards created by patients recovering from surgery. In particular, their model outputs the following ward-level statistics per day: 90th percentile occupancy, expected admissions, expected discharges, and expected numbers of patients in each day of recovery. This modelling work helped the Netherlands Cancer Institute-Antoni van Leeuwenhoek Hospital choose a new surgical block schedule after opening an extra operating room. The new schedule succeeded in smoothing the numbers of beds required on the ward compared to alternative schedules considered.

Two related studies focus on the scheduling of nurses in operating rooms (Lim, Mobasher, Bard, & Najjarbashi, 2016; Mobasher, Lim, Bard, & Jordan, 2011). Mobasher et al. (2011) develop a multi-objective mixed-integer program, with six soft constraints modelled as penalised objectives. The aim is to assign nurses to surgery cases taking account of their specialties and skills. Lim et al. (2016) extend this work by adding a second optimisation to maximise the number of nurses who can take breaks over lunchtime. Four different methods are proposed to solve the assignment problem; the swap heuristic and column generation approach proposed in later work (Lim et al., 2016) find acceptable solutions much more quickly than the earlier ones, a new version of modified goal programming and the solution pool method (Mobasher et al., 2011).

### 4.5. Chemotherapy scheduling

There are papers considering a variety of chemotherapy scheduling problems. Hahn-Goldberg et al. (2014) address the online scheduling problem: how to optimise a daily schedule of patients when new requests must be scheduled as they arrive. First a template of appointment times is created by optimising over a sample of previous appointment requests. As requests arrive they are assigned appointment times within this template, until this is no longer possible, when the schedule is re-optimised based on the requests that have arrived up to that point. The optimisations are solved using constraint programming to minimise the total working time (makespan) on a particular day. Finally, an algorithm to shift appointment times is applied to deal with cancellations. Using this approach the authors are able to improve the makespan compared to current scheduling practices by up to 20%.

The goals of the patient scheduling study by Santibáñez et al. (2012), on the other hand, are to notify patients of their appointment at least a week in advance and to reduce the waiting list size. In the first stage of the project at a cancer centre in British Columbia (Canada), a detailed process review of current booking practices and a patient survey were carried out. A discrete event simulation is developed to test changes to booking processes. Then a multi-objective optimisation program is presented which creates a daily appointment schedule including nurse allocation. This aims to satisfy patient preferences, balance the numbers and complexity of patients per nurse throughout the day, assign clinical trial patients to specialised nurses, and limit pharmacy workload dependent on available resources. Resulting from the modelling work, changes were made to booking practices and software for the optimisation tool was introduced. Following the changes, the median wait list size decreased, the numbers of patients notified of their appointment less than a week in advance decreased, and patient satisfaction increased.

Woodall, Gosselin, Boswell, Murr, and Denton (2013) also describe a discrete event simulation of a cancer centre, this one being in North Carolina (USA). The simulation enables the authors to identify the service bottleneck: nurses who administer chemotherapy to particular disease groups. Secondly, the authors develop a mixed-integer program to optimise the weekly and monthly schedules of different nurse types to minimise the hours of unmet demand. Thirdly, they use simulation-optimisation to determine the best nurse shift start times so that average patient waiting time is minimised. In particular, it was found that shift start times should be allowed to start on the half hour and that more nurses were needed. Consequently, the cancer centre adjusted shift times and hired more nurses. Additionally, the models were used to help plan staffing levels for a new cancer centre.

### 4.6. Radiotherapy scheduling

In this section, we focus on examples of papers that have addressed the pre-treatment scheduling and treatment scheduling problems. Vieira, Hans, van Vliet-Vroegindeweij, van de Kamer, and van Harten (2016) produced a more inclusive review on radiotherapy resource use that includes 18 papers on patient scheduling.

A study based in the Academic Medical Centre in Amsterdam offers potential improvements to radiotherapy treatment access times, which are the time from referral until treatment starts (Bikker, Kortbeek, van Os, & Boucherie, 2015). The authors develop an integer linear program to optimise when doctors should be allocated to pre-treatment tasks in order to minimise the access time for all patient types. In order to assess the impact of their schedules in a more realistic stochastic situation, they conduct experiments in a discrete event simulation model. On the other hand, Castro and Petrovic (2012) model the problem from the patient perspective. Pre-treatment appointments consist of multiple stages requiring different resources. They solve this scheduling problem as a hierarchy of optimisation problems with different waiting time objectives. Since this approach does not yield a feasible solution in a short enough time, they also experiment with six different rules to generate the initial solution to the first problem more quickly.

Several papers schedule patients for radiotherapy treatment, by determining what day each patient should start treatment given available LINAC capacity, and assuming the timing of continuing treatment follows a fixed pattern. Some of these tackle the offline problem (Petrovic, Morshed, & Petrovic, 2011), whereas Sauré, Patrick, Tyldesley, and Puterman (2012) and Legrain, Fortin, Lahrichi, and Rousseau (2015) solve the online problem, where appointment requests are scheduled as they arrive. Objective functions involve minimising days waiting to start treatment (Conforti, Guerriero, & Guido, 2010; Legrain et al., 2015; Petrovic, Morshed, et al., 2011; Sauré et al., 2012), days overdue to start treatment (Legrain et al., 2015; Petrovic, Morshed, et al., 2011), overtime (Legrain et al., 2015; Sauré et al., 2012) and booking decisions postponed (Sauré et al., 2012). All these papers consider patients with different priorities, for example, curative and palliative groups.

The authors of these treatment scheduling papers use a variety of approaches (Conforti et al., 2010; Legrain et al., 2015; Petrovic, Morshed, et al., 2011; Sauré et al., 2012). Conforti et al. (2010) provide an integer program and solve it using exact methods. Petrovic, Morshed, et al. (2011) compare the performance of different genetic algorithms to solve their multi-objective optimisation and find that the algorithm prioritising emergency patients performs best overall. Sauré et al. (2012) formulate the problem as a Markov decision process which is reformulated using approximate dynamic programming to obtain a linear program, for which the dual problem is solved using column generation. They simulate the generated scheduling procedure for a case study, and find that there would be improvements to waiting times compared to current practice. Legrain et al. (2015) first solve an offline optimisation problem using two algorithms. Then the uncertainty of treatment duration and new patients arriving is incorporated to produce a stochastic online optimisation. Algorithms to solve this are also presented. Results show that the model improves on current scheduling practice.

### 4.7. Chemotherapy treatment planning

Mathematical approaches to optimising chemotherapy plans have recently been reviewed by Shi, Alagoz, Erenay, and Su (2014), and the only paper our search found that is more recent than the scope of that review is by Alam et al. (2013). For a particular patient, chemotherapy treatment planning involves balancing two objectives: destroying as many cancer cells as possible while minimising the toxicity to normal cells (Cancer Research UK, 2016). An added complexity is that cancer cells may become resistant if exposed to drugs for a long enough time.

Shi et al. (2014) reports that many papers formulate the problem as an optimal control model and aim to shrink the tumours as much as possible over a fixed time period, given tumour growth rate and limits on the chemotherapy drug dose. These models are single or multi-objective optimisations that involve solving systems of differential equations. Researchers may consider dosages, at what time points to treat patients (for example cyclically or continuously) and single or multiple drugs. Since these differential equations are challenging to solve analytically, a range of approaches including simplifying the model then solving it exactly, approximations and heuristics have been applied (Shi et al., 2014). Some authors add even further complexity by modelling the problem stochastically to capture randomness in the rates of tumour growth and drug-induced shrinkage (Shi et al., 2014). In order to encourage the application of these models in clinical practice, Shi et al. (2014) recommend focusing on a specific cancer type, including cost as an objective, modelling how treatment plans are updated and only considering solutions that are feasible in practice.

Alam et al. (2013) provide a recent example of a multi-objective optimisation of chemotherapy plans, and consider multiple drugs. Compartment models are used to describe cancer cell change, where cells in different phases (resting, dividing, or dead) are affected by the drugs to a greater or lesser extent. The objectives are: reducing the numbers of both resting and dividing cancer cells, maximising the number of normal cells, reducing the toxicity and keeping the drug concentration within an acceptable limit. This sophisticated model is formulated as a closed-loop optimal control model and solved using a genetic algorithm. An extensive set of experiments with different numbers of drugs is carried out and comparisons to results of other models are made. Unfortunately, attention to Shi et al. ’s (2014) recommendations for improving practical relevance are not evident here, but the authors do perform robustness analysis.

### 4.8. Radiotherapy treatment planning

Here we describe OR approaches to planning different types of radiotherapy. First we discuss the papers on internal radiotherapy: low-dose rate brachytherapy, high-dose rate brachytherapy, and radioactive iodine. Then we discuss the papers on external radiotherapy: 3D conformal radiotherapy and intensity modulated radiation treatment (IMRT).

#### 4.8.1. Internal radiotherapy: low-dose rate brachytherapy

Lee and Zaider (2008) describe their extensive contributions to low-dose rate brachytherapy planning for prostate cancer (and reference their many earlier papers). In particular, their system enables the optimisation and re-optimisation of placement of radioactive seeds during the implantation procedure itself. This eliminates the need for patients to attend a planning appointment and associated scans. Their approach uses mixed-integer programming and a range of new solution methods involving “conflict hypergraphs” to deal with dense constraint matrices. The development and successful implementation of their system at the Memorial Sloan-Kettering Cancer Center was recognised with the presentation of the Franz Edelman Award. After implementation, the numbers of patients suffering side effects was substantially reduced, and procedures were shortened since fewer seeds were implanted. Ferrari, Kazareski, Laca, and Testuri (2014) build on Lee and Zaider ’s (2008) models and other models optimising the positioning of radioactive seeds. They add in extra constraints, for example constraints relating to the needles that position the seeds. Unlike Lee and Zaider (2008), their purpose is to optimise in advance of, rather than during, the procedure. Using a genetic algorithm to find acceptable solutions, they succeed in reducing the dose affecting surrounding organs compared to manually generated plans.

#### 4.8.2. Internal radiotherapy: high-dose rate brachytherapy

We refer interested readers to De Boeck, Beliën, and Egyed ’s (2014) review of dose optimisation models for high-dose rate brachytherapy between 1990 and 2010. These models optimise how long (dwell time) a radioactive source should stay in each position (dwell location). De Boeck et al. (2014) found that in the earlier papers, forward planning is the norm, where the dwell times are changed iteratively and the dose is calculated each time. These do not take into account the anatomy of particular patients. In later papers there is a move to inverse planning, where the desired dose is specified in advance, and images of individual patients’ anatomies are used in planning. Usually, the positions of the catheters containing the radioactive sources is given, but some papers also optimise these positions (De Boeck et al., 2014). The review found that models have been developed for a range of cancers, and both exact and heuristic methods have been used to solve them. De Boeck et al. (2014) categorise the multi-objective models depending on whether the importance of each objective is decided in advance, during or after the optimisation. It is recommended that future papers concentrate on making models more clinically relevant as well as incorporating uncertainty.

More recently, Lee et al. ’s (2013) work with the Rush University Medical Centre in the USA looked at the best dwell times and dwell locations for radioactive seeds in treating cervical cancer. This made use of new imaging technology called positron emission tomography to visualise where the cancer cells are densest, and increase the radioactive dose to these regions accordingly. A biological model was used to explicitly incorporate the probability of killing tumour cells in the objective function. The problem was modelled as a mixed-integer non-linear program. Heuristic solution methods were developed that made use of newly defined “generalised conflict hypergraphs” (see also the description of Lee et al. ’s (2013) work in the “Internal radiotherapy: low-dose rate brachytherapy” section). The mathematical model developed was used to plan treatment for patients involved in a clinical trial, and the trial was subsequently extended due to its success.

Recent work on high-dose rate brachytherapy for prostate cancer applies mixed-integer programs to optimise both the catheter positions and the dwell times (Holm, Carlsson Tedgren, & Larsson, 2016). The computations are done while the patient is under anaesthetic following 3D imaging and before treatment starts, so must be relatively quick. Therefore heuristics are used to find solutions in under an hour. It is found that variable neighbourhood search gives a better solution than tabu search, genetic algorithm, and CPLEX (a commercial solver) in this time frame.

#### 4.8.3. Internal radiotherapy: Radioactive iodine

Teodorović, Šelmić , and Mijatović-Teodorović (2013) consider an internal treatment for well-differentiated thyroid cancers, called radioactive iodine treatment. Unlike the above papers which aim to improve on current treatment plans, their goal is to replicate experienced physicians’ dose plans in order to train less experienced physicians. For this, they use a technique called case-based reasoning. This involves finding the most similar patient who has been treated in the past, and applying the same dose to the new patient. There are a range of ways in which patients may be similar, for example age, diagnosis and size of tumour. The authors optimise the importance of each of these similarity measures in order to most closely match the real doses that were applied with the doses predicted by the model. This is achieved using a Bee Colony Optimisation meta-heuristic.

#### 4.8.4. External radiotherapy: 3D conformal radiotherapy

Here we discuss some 3D conformal radiotherapy planning models (Obal, Volpi, & Miloca, 2013; Petrovic, Khussainova, & Jagannathan, 2016; Petrovic, Mishra, & Sundar, 2011). Petrovic, Mishra, et al. (2011) and Obal et al. (2013) model prostate cancer treatment, while Petrovic et al. (2016) apply their approach to brain cancer treatment. The decision variables are different in each paper. Petrovic, Mishra, et al. (2011) and Obal et al. (2013) aim to determine the appropriate dose. For Obal et al. (2013), this is the dose intensity that each beam should deliver over the whole treatment course, but for Petrovic, Mishra, et al. (2011) this is the total dose that should be delivered during phase 1, when both the prostate and surrounding area are targeted, and the total dose in phase 2, when only the prostate is targeted. Petrovic et al. (2016) decide on the appropriate number of beams and the beam angles.

Both Petrovic, Mishra, et al. (2011) and Petrovic et al. (2016) present case-based reasoning techniques that make use of previous treatment plans developed by medical experts. Petrovic, Mishra, et al. (2011) combine previous similar cases by taking into account how successful each treatment plan was (according to the prostate-specific antigen value measured two years after treatment) and using Dempster–Shafer theory. Simulated annealing is used to weight the features of cases. Petrovic et al. (2016) present methods to adapt previous cases without needing expert knowledge: neural networks, naïve Bayes classifier and adaptation-guided retrieval. On the other hand, Obal et al. (2013) formulate their model as a multi-objective linear program and use the weighted sum method to find the set of efficient solutions.

The authors present promising model results (Obal et al., 2013; Petrovic, Mishra, & Sundar, 2011; Petrovic et al., 2016). For most patients, the dose plans produced by Petrovic, Mishra, et al. (2011) were at least as good as those generated by oncologists, in that they met dose constraints and the total dose was at least as high. Similarly, Petrovic et al. (2016) defined the success rate as the number of plans that were the same as those designed by oncologists. In this context, using neural networks and adaptation-guided retrieval improved the specification of the number of beams compared to no adaptation, however the specification of beam angles was not improved by adaptation. In order to choose between the many efficient solutions to their problem, Obal et al. (2013) suggest choosing the solution nearest the “ideal point”, the point with the best solution for each objective.

#### 4.8.5. External radiotherapy: Intensity modulated radiation therapy (IMRT)

There is a large body of literature on designing IMRT plans and several review papers (Bortfeld, 2006; Censor & Unkelbach, 2012; Ehrgott, Güler, Hamacher, & Shao, 2010). Bortfeld (2006) discusses the mathematical, physical, and technological developments relating to IMRT. The author describes the typical problem formulation, which is to calculate the necessary beamlet intensities given prescribed doses that should reach the tumour site (target). This is known as inverse planning. Later Ehrgott et al. (2010) reviewed optimisation approaches to three related problems: (1) fluence map optimisation, which consists of finding the best set of beamlet intensities, (2) beam angle optimisation, and (3) the segmentation problem, which is how to configure the multileaf collimators to achieve this. Censor and Unkelbach (2012) describe the two key approaches to solving the inverse problem: continuous analytic techniques and fully discretised algebraic methods. They explain the change in perspective to considering the problem as an optimisation where the damage to healthy tissue should be minimised.

**Table 6.** Performance measures for each problem area.

Area	Performance measures	Papers
Reducing cancer risks	Technical merit of projects	Hall et al. (1992)
	Avoided cervical cancer cases and deaths, disability-adjusted life years, cost-effectiveness	Kim et al. (2013)
Screening strategies	Mortality measures	Brailsford et al. (2012), Tejada et al. (2014), Arrospide et al. (2015), Madadi et al. (2015), Wang and Zhang (2017) and McLay et al. (2010)
	Quality-adjusted life year measures	Ayer et al. (2012), Tejada et al. (2014), Madadi et al. (2015), Ayer (2015), Ayer et al. (2016), Erenay et al. (2014), Li et al. (2014), Li et al. (2015), Rauner et al. (2010) and Bertsimas et al. (2016)
	Cost-effectiveness measures	Tejada et al. (2014), O’Mahony et al. (2015), Song and Wang (2016)
	Total cost	Li et al. (2014) and Rauner et al. (2010)
	Cancer incidence	McLay et al. (2010)
	Overdiagnosis	Arrospide et al. (2015)
	False-positives	Arrospide et al. (2015) and Ayer et al. (2012)
	Number of mammograms	Ayer et al. (2012)
	Cancers detected	Brailsford et al. (2012)
Locating screening facilities	Uptake of screening	Haase and Müller (2015)
	Efficiency (fairness) and coverage	Gu et al. (2010)
Evaluating process changes to screening services	Waiting times	Pilgrim and Chilcott (2008)
	Overdue screenings	Zai et al. (2014)
Following up screening tests	Quality-adjusted life year measures	Chhatwal et al. (2010) and Alagoz et al. (2013)
Other screening-related studies	Waiting time, idle time and overtime	Baker and Atherill (2002)
	Tumour doubling times	Vieira et al. (2011)
Managing diagnostic resources	Total cost	Lee et al. (2014) and Örmeci et al. (2016)
	Mortality rate, cancer incidence rate	Güneş et al. (2015)
	Waiting time, overtime, revenue	Berg et al. (2013)
	Throughput, resource utilisation	Berg et al. (2010)
Optimising diagnostic procedures	Probability of detecting cancer	Sofer et al. (2003)
Staging accuracy	Expected information value of test combinations	Ekaette et al. (2006)
Treatment decisions	Survival benefit	Utley et al. (2006) and Simon (2009)
	Quality-adjusted life year measures	Simon (2009)
	Deciding on performance measures	Suner et al. (2012)
Access to treatment	Total demand-weighted distance	Cotteels et al. (2012)
	Total distance travelled	Chahed et al. (2009)
Performance of cancer treatment centres	Deciding on performance measures	Santos et al. (2007)
	Waiting time, closing time and resource utilisation	Baesler and Sepúlveda (2001)
	Total treatment planning time	Werker et al. (2009)
Surgery scheduling	Co-availability of staff	Mutlu et al. (2015)
	Ward cccupancy	Vanberkel et al. (2011)
	Nurse breaks	Mobasher et al. (2011) and Lim et al. (2016)
	Nurse overtime, nurse job changes, nurse room changes	Mobasher et al. (2011)
Chemotherapy scheduling	Total working time	Hahn-Goldberg et al. (2014)
	Balanced nurse workload, satisfying patient preferences, pharmacy workload limited, specialist nurses assigned appropriately	Santibáñez et al. (2012)
	Waiting time measures	Santibáñez et al. (2012) and Woodall et al. (2013)
	Demand satisfaction	Woodall et al. (2013)
(*Continued*)		

**Table 7.** (*Continued*).

Area	Performance measures	Papers
Radiotherapy scheduling	Access times	Bikker et al. (2015)
	Waiting time measures	Castro and Petrovic (2012), Conforti et al. (2010), Petrovic, Morshed, et al. (2011), Sauré et al. (2012) and Legrain et al. (2015)
	Overtime	Sauré et al. (2012) and Legrain et al. (2015)
	Booking decisions postponed	Sauré et al. (2012)
Chemotherapy treatment planning	Number of cancer cells remaining (resting and dividing), number of normal cells remaining, toxicity, drug concentration	Alam et al. (2013)
Radiotherapy treatment planning	Deviations from prescribed radiation doses in tumour, surrounding organs and normal tissue	Lee and Zaider (2008) and Ferrari et al. (2014)
	Number of needles, number of radioactive seeds	Ferrari et al. (2014)
	Deviations from prescribed doses in tumour, surrounding organs and normal tissue	Lee and Zaider (2008), Petrovic, Mishra, et al. (2011), Bertsimas et ail (2013), Chan and Mišić (2013), Lee et al. (2013), Obal et al. (2013), Aleman et al. (2014), Cabrera et al. (2014), Chan et al. (2014), Dias et al. (2014), Ferrari et al. (2014), Holm et al. (2016), Mahmoudzadeh et al. (2016), Van Haveren et al. (2017)
	Tumour control probability	Lee et al. (2013)
	Difference to expert-generated treatment plan	Teodorović et al. (2013) and Petrovic et al. (2016)
	Total dose	Petrovic, Mishra, et al. (2011) and Chan and Mišić (2013)
	Numbers of cancer cells and normal cells killed	Jalalimanesh et al. (2017)
	Deviation between planned and actual dose	Chan et al. (2014)
	Number of apertures used	Taşkin and Cevik (2013)
Other treatment-related studies	Deviations from prescribed doses in tumour, critical regions and normal tissue	Holder and LLagostera (2008)
	Deciding criteria for drugs to produce in advance	Vidal et al. (2010)
	Waiting times	Masselink et al. (2012)

**Table 8.** Table showing numbers of papers identified in each area.

Cancer service	Problem	Subproblem	Review papers	Identified papers (since latest review)
Prevention	Reducing cancer risks			2
	Screening strategies		6 reviews, latest published in 2011	19
	Locating screening facilities			2
	Evaluating process changes			2
	Following up screening			2
	Other screening			2
Cancer diagnosis and staging	Managing diagnostic resources			5
	Optimising diagnostic procedures			1
	Staging accuracy			1
Treatment	Treatment decisions			3
	Access to treatment			2
	Performance of cancer treatment centres			4
	Surgery scheduling			4
	Chemotherapy scheduling			3
	Radiotherapy scheduling			6
	Chemotherapy treatment planning		1 review, published in 2014	1
	Radiotherapy treatment planning	Low-dose rate brachytherapy		2, 1 of which also references 10 earlier related papers by the authors
		High-dose rate brachytherapy	1 review, published in 2014	2
		Radioactive iodine		1
		3D conformal		3
		IMRT	3 reviews, latest published in 2012	10
	Other treatment-related studies			3

We compare examples of IMRT treatment planning papers published since Censor and Unkelbach ’s (2012) paper that were identified from our search strategy. Some of these address the fluence map optimisation problem (Cabrera, Ehrgott, Mason, & Philpott, 2014; Chan, Mahmoudzadeh, & Purdie, 2014; Mahmoudzadeh, Purdie, & Chan, 2016; Van Haveren et al., 2017). Chan and Mišić (2013) and Aleman, Wallgren, Romeijn, and Dempsey (2014) optimise fluence maps over the whole series of treatment sessions, called fractions. Aleman et al. (2014) consider the case where there are two target areas that require differing amounts of radiation. Jalalimanesh, Haghighi, Ahmadi, and Soltani (2017) optimise the number of fractions and dose per fraction, rather than assuming constant doses as is commonly the case. Dias, Rocha, and Ferreira (2014) address the beam angle optimisation problem, while Bertsimas, Cacchiani, Craft, and Nohadani (2013) optimise both angles and intensities jointly. The “leaf sequencing” problem is considered by Taşkin and Cevik (2013): which sequence of rectangular aperture shapes and intensities to use so that the total planned intensities are achieved.

There may be as many as 30 objectives to consider in radiotherapy planning optimisation (Van Haveren et al., 2017). Importantly, the tumour should receive enough radiation, called target coverage, while normal tissues and surrounding organs should receive as little as possible, called organ sparing. For example, some models minimise the “conditional value-at-risk”, which is the average dose that is received in the parts of an organ that receive the highest dose (Chan et al., 2014; Mahmoudzadeh et al., 2016). Different optimisation, heuristic and simulation methods have been used on these problems. Van Haveren et al. (2017) solve their multi-objective optimisation by applying the lexicographic reference point method and Cabrera et al. (2014) prove theoretical results for solving a particular class of multi-objective optimisations through a series of single-objective optimisation problems. Aleman et al. (2014) penalise deviations from the required dose in their optimisation, and solve it using a primal-dual interior point algorithm. Taşkin and Cevik (2013) use combinatorial Benders decomposition to break down their mixed-integer program into an integer program master problem and a linear program subproblem, then compare heuristic and exact solution procedures. Bertsimas et ail (2013) develop a heuristic combining simulated annealing with gradient descent to solve their linear program. On the other hand, Dias et al. (2014) provide a non-linear formulation and find solutions with a genetic algorithm incorporating a neural network to estimate the fitness functions quickly. Jalalimanesh et al. (2017) develop an agent-based simulation of tumour growth and use the Q-learning algorithm, a type of reinforcement learning, to optimise. A series of papers use robust optimisation to capture the uncertainty in patients’ breathing patterns (Chan & Mišić, 2013; Chan et al., 2014; Mahmoudzadeh et al., 2016). Chan and Mišić (2013) update what is known about a patient’s breathing each treatment to help with planning the next treatment. This is modelled as a series of linear programs. Mahmoudzadeh et al. (2016) use constraint generation (a decomposition method) to solve the robust optimisation first presented by Chan et al. (2014).

Some authors report how their methods improved solutions or computational time compared to other approaches (Aleman et al., 2014; Chan & Mišić, 2013; Dias et al., 2014; Taşkin & Cevik, 2013; Van Haveren et al., 2017). Mahmoudzadeh et al. (2016) found that adding one constraint each time their problem was resolved was slowest, and it was fastest to add several constraints each time. Compared to the standard treatment planning method at the time, Chan et al. ’s (2014) approach better matches the planned dose with the actual dose received, since they incorporate breathing uncertainty. Jalalimanesh et al. (2017) show that the dose should be varied over time as the tumour changes size. Cabrera et al. (2014) demonstrate how to generate infinitely many Pareto-optimal solutions to their multi-objective optimisation problem.

### 4.9. Other treatment-related studies

Holder and LLagostera (2008) model how best to apply photodynamic therapy to deep tissue cancers, and assess whether the only drug approved in the USA for this treatment at the time would yield acceptable results. They develop biological models of the concentration of the drug in different tissues, and the rate at which cellular damage occurs when the drug is activated with light. Their linear model to optimise the application of the light source is adapted from a model for external radiotherapy planning. They find that even under the optimal alignment of the light source, the drug cannot target the tumour closely enough, and the surrounding normal tissues are damaged to an unacceptable extent.

Vidal, Sahin, Martelli, Berhoune, and Bonan (2010) and Masselink, van der Mijden, Litvak, and Vanberkel (2012) both address the decision of which chemotherapy drugs to produce in advance, rather than on demand directly before use. These drugs are prepared for specific patients, and so become useless if patients are too ill for treatment and the drugs expire before they can be used. Vidal et al. (2010) interviewed pharmacists at a French pharmacy, which prepares drugs for medical facilities in the area, to find out criteria that make a drug suitable for preparing in advance. The relative importance of each criterion was determined using the analytical hierarchy process. The most important criteria were found to be drug stability, time between ordering and when needed, as well as total annual volume of the drug. Following the study, a decision support tool, designed to assist in choosing the drugs to produce in advance, was adopted by other pharmacies in France.

Masselink et al. (2012) worked with a pharmacy that is attached to a chemotherapy unit in the Netherlands. Unlike Vidal et al. (2010), they focus on the effect on patient waiting times of preparing some chemotherapy drugs in advance. This is achieved by modelling the drug order queue as well as the linked queue of patients waiting for treatment. For the case study, a discrete event simulation is developed. Analytical expressions to approximate patient waiting times under different policies are derived, which are also applicable to other settings. The policies considered involve making the cheapest drugs (up to some threshold price) in advance and optionally reallocating drugs when patients are too ill for treatment. Using the results of the modelling work, managers from the pharmacy and chemotherapy unit agreed on which drugs should be made in advance and that some drugs should be reallocated. Model results suggest this will cause waiting times to be halved with only a 1–2% increase in cost.

## 5. Conclusion and discussion

Our review showcases examples of OR techniques applied to problems throughout cancer care services. These problems lend themselves to OR modelling because of conflicting objectives, large numbers of options to be compared and patient-specific parameters. A key strength of many OR methods is that they can make goals, constraints, and uncertainties explicit. Using our search strategy (described in the Introduction section), a substantial amount of research on screening strategies was discovered, as well as on treatment planning and scheduling. Arguably these areas are particularly suited to OR modelling, since they have the characteristics described at the start of this paragraph. On the other hand, we uncovered comparatively few examples of OR models applied to reducing cancer risks, optimising diagnostic procedures and staging. Sourcing appropriate data may be challenging when assessing population-level interventions to reduce cancer risks, and optimising diagnostic procedures and staging are more typically considered in clinical trials. The numbers of papers identified for each problem area are summarised in Table 6.

This review is not systematic; in particular it only includes papers that mention “operations research” or “operational research”. This means that there are further published applications to cancer care which use techniques that are considered OR methods. However, this paper goes some way towards demonstrating the huge variety of cancer care problems that have benefited from an OR perspective. We also hope that reading this paper will prove a useful starting point for OR researchers considering tackling cancer care problems.

It is promising that there is evidence of improvements being made throughout cancer services as a result of operational research modelling. At the national or regional level, OR studies have influenced decisions on screening strategies (Koleva-Kolarova et al., 2015), cervical screening processes (Pilgrim and Chilcott, 2008) and funding of anti-cancer proposals (Hall et al., 1992). At the hospital or department level, OR studies have impacted brachytherapy delivery (Lee & Zaider, 2008), surgical schedules (Vanberkel et al., 2011), chemotherapy booking practices and schedules (Santibáñez et al., 2012), nurse hiring and shift start times (Woodall et al., 2013) and cancer treatment centre capacity, layout and scheduling processes (Matta & Patterson, 2007). Additionally, Simon (2009) developed a treatment decision aid that is accessible online. On the other hand, many theoretically impressive studies fail to translate into service changes, or at least implementation is not evident from the papers, as Shi et al. (2014) also point out for the specific case of chemotherapy treatment planning. There is potential for closer working with clinicians to enable the impact of these methods to be of greater benefit to cancer sufferers. When targeting implementation, it is crucial that methods are transparent and that appropriate performance measures are chosen.

Finally, we outline some opportunities for future research. Since the number of cancer cases is increasing, in part due to lifestyle factors, there is scope to prevent a large number of cancer cases, with huge potential benefits for both would-be patients and the health budget. OR techniques, for example pathway modelling, are well suited to assess the potential impact of different prevention schemes, such as incentives for lifestyle changes. At the same time, it is important to understand the issue of overdiagnosis better in order to prioritise limited resources appropriately and avoid unnecessary worry, tests, and treatment (Moynihan, Doust, & Henry, 2012). Mathematical modelling of the impacts of overdiagnosis could help. In terms of cancer survival, the UK lags behind its western European counterparts (De Angelis et al., 2014). More research could be done looking at what differences between national care systems cause this discrepancy and what would happen if processes from other countries were adopted in the UK. To this end, simulation models for example could be valuable. OR methods such as case-based reasoning, which is used in treatment planning, may require large data-sets in order to provide the best solutions. Thus for the rarer cancer types it may help to pool national or even international data-sets. We believe these are worthwhile research areas for those keen to contribute to the OR literature on cancer care.
